# Assessment of semi-quantitative parameters for visual interpretation of stress-perfusion CMR in obstructive coronary artery disease

**DOI:** 10.1186/1532-429X-15-S1-P210

**Published:** 2013-01-30

**Authors:** Christoph J Jensen, Lowie M Van Assche, Deneen Spatz, Michele Parker, Wolfgang G Rehwald, Raymond J Kim, Igor Klem

**Affiliations:** 1 Duke Cardiovascular Magnetic Resonance Center, Duke University, Durham, NC, USA; 2 Siemens Healthcare, Chapel Hill, NC, USA

## Background

Adenosine stress CMR with visual interpretation is increasingly used in the evaluation of patients with CAD. The definition of a stress perfusion defect is inconsistent in the literature regarding i) the duration from contrast arrival, ii) persistence relative to a remote segment, iii) transmural extent, and iv) reversibility on rest perfusion.

In this study we sought to test several semi-quantitative parameters assessed by rapid visual analysis, and determine their utility to identify stress-perfusion defects from obstructive CAD.

## Methods

25 patients (61±14 years, 44% male) with known CAD and ≥70% stenosis on invasive coronary angiography (CA) were studied (60% single-vessel, 40% two-vessel disease). All patients underwent CMR within 2 months of CA, typically, four short-axis images were obtained each heartbeat during adenosine stress and rest. Patients with infarction, revascularization, and cardiomyopathy were excluded.

Stress and rest perfusion images were analyzed visually using a 16-segment model. The number of frames from left-ventricular (LV) cavity peak contrast to myocardial signal homogeneity (MET) within each segment was assessed (n=400 segments). We scored the transmural extent (in quartiles) of any segmental hypoenhancement persiting ≥1 frame beyond the first homogenous segment in the same slice. CA was reviewed to determine for each segment, whether they are subtended by a stenotic coronary artery ("ischemic" vs "non-ischemic" segments).

## Results

**Figure 1:**
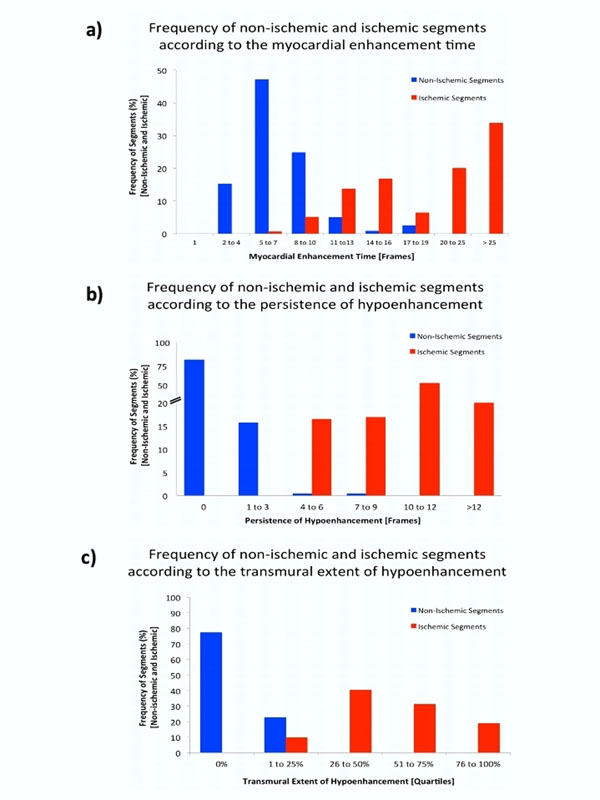
Results of different semi-quantitative parameters (Myocardial Enhancement Time, Persistence of Hypoenhancement, Transmural Extent of Hypoenhancement) derived from the visual interpretation of Stress-Perfusion CMR. The frequency of non-ischemic and ischemic segments in each group is expressed as percent of the total number of non-ischemic or ischemic segments, respectively. A) Frequency of non-ischemic (blue) segments and ischemic (red) segments according to groups of different Myocardial Enhancement Times (x-axis) in frames. B) Frequency of non-ischemic (blue) segments and ischemic (red) segments according to the Persistence of Hypoenhancement (x-axis) in frames. C) Frequency of non-ischemic (blue) segments and ischemic (red) segments according to the Transmural Extent of Hypoenhancement (x-axis) in quartiles.

The input function (number of frames from LV contrast arrival to LV peak contrast enhancement) ranged between 4-7 frames (median 5, IQR 4-6) in all patients. There were 154 ischemic and 246 non-ischemic segments. Nine non-ischemic segments had a matched rest perfusion defect (e.g. artifactual), none of the ischemic segments showed a rest perfusion defect. MET for ischemic segments was longer (median 22, interquartile range [IQR] 14-30) than in non-ischemic segments (7, IQR 5-8, p<0001), however, 38% of segments overlapped (Figure 1a). Most non-ischemic segments were concurrently homogenous (0, IQR 0-0), in two segments hypoenhancement persisted for >3 frames, both had matched rest perfusion defects (Figure 1b). In ischemic segments hypoenhancement persisted for a median of 14 (IQR 8-22) frames (p<0.0001), with a minimum of 4 frames. The transmural extent of hypoenhancement (Figure 1c) was higher in ischemic segments (3, IQR 2-3) compared to non-ischemic segments (0, IQR 0-0], 139 (90.3%) ischemic segments were >25% transmural.

## Conclusions

Stress-perfusion defects in obstructive CAD tend to be >25% in transmural extent, persist for >4 frames, and have no corresponding rest perfusion defects.

## Funding

none

